# Applying the functional abnormality ontology pattern to anatomical functions

**DOI:** 10.1186/2041-1480-1-4

**Published:** 2010-03-31

**Authors:** Robert Hoehndorf, Axel-Cyrille Ngonga Ngomo, Janet Kelso

**Affiliations:** 1 Institute for Medical Informatics, Statistics and Epidemiology, University of Leipzig, Leipzig, Germany; 2 Department of Computer Science, University of Leipzig, Leipzig, Germany; 3 Department for Evolutionary Genetics, Max Planck Institute for Evolutionary Anthropology, Leipzig, Germany; 4 European Bioinformatics Institute, Wellcome Trust Genome Campus, Hinxton, Cambridge, CB10 1SD, UK

## Abstract

**Background:**

Several biomedical ontologies cover the domain of biological functions, including molecular and cellular functions. However, there is currently no publicly available ontology of anatomical functions.

Consequently, no explicit relation between anatomical structures and their functions is expressed in the anatomy ontologies that are available for various species. Such an explicit relation between anatomical structures and their functions would be useful both for defining the classes of the anatomy and the phenotype ontologies accurately.

**Results:**

We provide an ontological analysis of functions and functional abnormalities. From this analysis, we derive an approach to the automatic extraction of anatomical functions from existing ontologies which uses a combination of natural language processing, graph-based analysis of the ontologies and formal inferences. Additionally, we introduce a new relation to link material objects to processes that realize the function of these objects. This relation is introduced to avoid a needless duplication of processes already covered by the Gene Ontology in a new ontology of anatomical functions.

**Conclusions:**

Ontological considerations on the nature of functional abnormalities and their representation in current phenotype ontologies show that we can extract a skeleton for an ontology of anatomical functions by using a combination of process, phenotype and anatomy ontologies automatically. We identify several limitations of the current ontologies that still need to be addressed to ensure a consistent and complete representation of anatomical functions and their abnormalities.

**Availability:**

The source code and results of our analysis are available at http://bioonto.de.

## Background

The notion of *function *is important throughout biology. It is used to characterize biological sequences [1], cell types [2], anatomical structures [3] and to annotate gene products [4]. Functions are also used in the description of phenotypes of *functionings*, i.e., observable phenomena regarding the functioning or malfunctioning of biological entities. These phenotypes play an important role in the discovery of gene functions and in the description of abnormalities, diseases, signs and symptoms.

### Phenotype ontologies

We define a phenotype as any observable characteristic of an organism, part of an organism or process in which an organism or one of its parts is involved. Phenotypes may include both structural and behavioral properties. *Functional phenotypes *are either observable characteristics of a process that realizes a function of an organism or a part of the organism, or properties of an organism that involve its functions (such as having a function or lacking a function).

Phenotype ontologies for mouse and human phenotypes were developed to annotate research databases of mouse and human phenotypes. The Mammalian Phenotype Ontology (MPO) focuses on mutant mouse phenotypes [5] and the Human Phenotype Ontology (HPO) focuses on Mendelian diseases in man [6]. They make an explicit reference to anatomy ontologies in their cross-product definitions [7], and implicit reference to the anatomy ontologies in the naming of their categories.

The HPO uses the Foundational Model of Anatomy (FMA) [3] to refer to anatomical entities in humans, and the MPO uses the Adult Mouse Anatomy Ontology (MA) [8]. These anatomy ontologies describe anatomical entities by using, among others, part-whole relations, i.e., they focus on the anatomical *structure*.

Although the phenotype ontologies describe both structurally and functionally abnormal phenotypes, the anatomy ontologies do not include an elaborate description of the anatomical functions. As a consequence, although the classification of structural abnormalities in the phenotype ontologies follows well-defined principles, the classification of phenotypes of functionings is often unprincipled and sometimes ambiguous. To address the issue of representing functional phenotypes, we provide an ontology design pattern [9] for functional abnormalities. This design pattern is applicable in phenotype ontologies, especially in the MPO and HPO. We discuss the benefits of the application of the design pattern and relate the design pattern to the composite names of the categories in the phenotype ontologies. Based on the category names, we apply a pattern-based approach to extract a skeleton for an ontology of anatomical functions from a combination of the anatomy and phenotype ontologies together with the Biological Process ontology of the Gene Ontology (GO) [4].

### Biological functions

There is an ongoing discussion in the philosophy of biology and theoretical biology as to the exact nature of a biological function. While functions of artifacts come into being due to the intentions of a designer, biological entities have evolved over time, and biological functions are not dependent on intentions in the same way as artifacts are.

Philosophical theories of biological functions range from reductions to causality over social accounts of functions to the denial of the existence of biological functions. The first two are of major importance, i.e., the causal view of biological functions and the social view of biological functions. The major proponents of causal explanations of functionality are Wright [10] and Millikan [11], while the social view is defended by Searle [12].

Wright gives the following definition of function [10]:

**Definition 1**. *"The function of X is Z" means*

1. X is there because it does Z,

*2. Z is a consequence (or result) of X's being there*.

In the definition, *X *is a category of structures and *Z *is a process category, and instances of *X *are involved in instances of *Z*. In its definition, Wright does not distinguish between functions and the processes which realize the function. Furthermore, the definition assumes that an entity has only one function. However, as discussed by Wright [10], the definition can be restated for entities having multiple functions by replacing "the function of *X *is *Z*" with "*a *function of *X *is *Z*".

**Figure 1:**
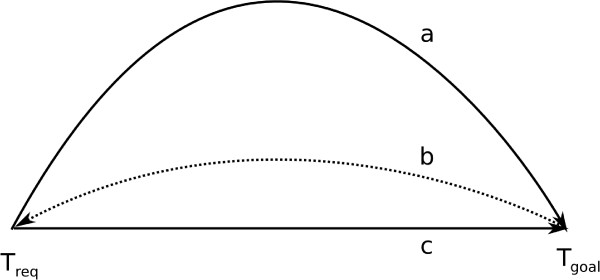
**Three steps for function ascription**. The figure shows the three conditions for the ascription of *a single *function to an entity. First, the goal of the function is established *in the future*. Second, the means for achieving the goal are selected or created. Finally, the goal can be realized by causal means, i.e., without the need for accessing or anticipating future states of the world.

In the social view, functions are ascribed to *brute facts *by a conscious observer [12]. A detailed analysis is provided by Hartmann [13] and is illustrated in Figure 1. Hartmann distinguishs three elements to the ascription of a function: the setting of a goal in the future, the planning of how to achieve the goal, resulting in a *structure *that is capable of achieving the goal through causal means. Figure 1 shows how some entity obtains a single function, according to Hartmann [13]. For an entity to have multiple functions, the same three steps are performed, yet the goal and the initial situation may change.

## Methods

### Ontology of functions

We do not choose a particular definition of biological function, and we do not add another definition to the literature. The method we present is compatible with most major views of *function*.

An analysis of how to represent functions has been provided by the Ontology of Functions (OF) [14,15]. A function in the OF is described in terms of a requirement situation type, a goal situation type and a processual role. The requirement situation type serves as precondition for any function realization, the goal situation type is the postcondition, and the processual role [16] is used to describe *how *a function bearer brings about the goal from the requirements. One major advantage of the treatment of functions in the OF is the explicit inclusion of preconditions for the function realizations, which serve to model the contexts in which a function can be realized.

### Function realizations

Functions can be *realized *multiple times. Each realization of a function is a process, and in each realization of a function the function bearer achieves the goal of the function, starting at a situation satisfying the preconditions of the function.

While a function is an entity that is similar to a property in that it inheres in its bearer, a *functioning *is a process that is a realization of a function. For example, while the function of the heart "to pump blood" is a property that the heart has in virtue of being a heart and in virtue of the evolutionary history of hearts, a *functioning *is the actual process of pumping blood which realizes the function of the heart [17]. In particular, the function of the heart is "to pump blood" even when the heart is not *functioning*. This could be the case during a heart transplantation or during a malfunctioning of the heart.

Furthermore, the function of the heart is "to pump blood" even when the heart *cannot *realize this function. Function realizations always require a *disposition *to realize the function in the function bearer, while the function itself can exist without such a disposition. For our present work, we use the simple conditional analysis of dispositions [18]: "something *x *is disposed at time *t *to give response *r *to stimulus *s*, iff, if *x *were to undergo *s *at *t*, *x *would *r*".

There are other possible causes for a heart to not function, e.g., an abnormality in the nervous system. This is not a malfunctioning of the heart. We define a function by its preconditions and postconditions. In the case that the nervous system fails, a precondition of the heart's function is not satisfied. If this precondition was satisfied, the heart would in fact pump blood (assuming the heart is functional).

Therefore, it is not the heart's being malfunctional but rather a non-satisfied precondition that causes the heart to not function. Within a wider context, i.e., the whole body, this may appear to be a malfunctioning of the heart, but the original cause was elsewhere - in the nervous system. The heart is functional, the nervous system abnormally functioning. A repair or treatment of such a condition should treat the nervous system and not the heart.

### Abnormal functionings

Abnormal functionings are processes which are similar to a functioning, but which are impaired in some way. We distinguish between abnormal functionings and *malfunctionings*: in the case of a malfunctioning, the function bearer cannot cause the goal of its function although the preconditions for a function realization are given. An entity *e *has the property of being *malfunctional *(with respect to the function *f*), if *e *has a function *f*, but not a *disposition d *to realize the function *f *. Functions and dispositions are disjoint categories (i.e., neither is a subcategory of the other), yet they are related in a particular way [19]. While abnormal functionings are processes, being *malfunctional *is a property of the function bearer; in the case of a malfunctional entity, no process of functioning can occur.

There are various kinds of abnormal functionings: functionings may be more or less effective, have unwanted side-effects or similar. We focus on the *malfunctional *property here. A classification of kinds of abnormal functionings is out of the scope of this paper and will be subject to future work.

### Function and Structure

There is an important relationship between *function *and *structure*. Biological functions are usually realized through causal processes (cf. [13] and Figure 1) and the function bearer has developed through evolution to play a particular role in processes of a certain kind (e.g., the role of the heart as a *pump *in its function *to pump blood*). Therefore, if the heart - the function bearer - becomes *unable *to play this role in the function realization, while everything else remains unchanged, this loss of disposition is due to a change in the heart's structure. In general, the loss of a disposition in the case of malfunctional entities must go along with a change in the structure of the bearer of the disposition and function.

As a result, if *e *has a biological function *f*, and *e *is malfunctional with respect to *f*, then *e *is abnormal. This pattern is already implied in the taxonomic backbones of the phenotype ontologies and reflected in the naming and the definitions of the phenotype ontologies' categories.

### The functional abnormality pattern

The functional abnormality pattern is an ontology design pattern [9] for ontologies that classify both abnormal structural and functional phenotypes, such as both the Human and Mammalian Phenotype Ontology.

According to the functional abnormality pattern, an abnormality of functioning (a property of a process) *implies *an abnormality of the function bearer (provided that external circumstances are normal). If multiple types of entities have the same kind of function, then an abnormality of the functioning *implies *a disjunction of the abnormalities of each possible kind of function bearer. On the other hand, being *malfunctional *(a property of the function bearer) is *a sub-category of *(**is-a**) an abnormality of the function bearer, and if multiple types of entities have the same function, then being *malfunctional *is *a sub-category of *a disjunction of the abnormalities of each possible kind of function bearer.

For example, an abnormality in *HearingP *processes (we use *HearingF *to refer to the function, and *HearingP *to refer to the process realizing the function; *HearingP *processes are functionings of the *HearingF *function) implies an abnormality of the ears, if the function of the ears is *HearingF *(and only the ears have the function *HearingF*). If the function of both the left ear and the right ear was *HearingF*, then an abnormality of *HearingP *implies an abnormality of the left ear or an abnormality of the right ear. In this case, the category "abnormality of the left ear or abnormality of the right ear" should be named "ear abnormality" and defined as a disjunction of the two categories "abnormality of the left ear" and "abnormality of the right ear", which are both sub-categories of "ear abnormality".

On the other hand, the ears' being *malfunctional *with respect to their *HearingF *function is a property of the ears, and should be classified as a sub-category of *Ear abnormality*. The ears' being *malfunctional *is defined as the absence of a disposition which would normally be present (due to the ears' having a function whose realization requires the disposition), and the loss of a disposition entails a structural modification according to the theory of dispositions [18]. Therefore, a loss of a disposition is a special kind of structural change of the disposition's bearer.

### Naming patterns in the phenotype ontologies

Our goal is to represent functional phenotypes formally. While there is no ontology of anatomical functions yet, such an anatomical function ontology is implied in the phenotype ontologies. These ontologies classify abnormal phenotypes, and in these phenotype ontologies, abnormal functionings are usually classified as a sub-category of abnormal structures which bear the function that is impaired. Therefore, the phenotype ontologies can serve as a seed for the construction of an ontology of anatomical functions.

However, as the phenotype ontologies rarely define abnormal functionings formally, the challenge is to extract the information about anatomical structures and their functions from the current ontology structure, category names and definitions of the phenotype ontologies. Such an approach will be insufficient to create an exhaustive ontology of anatomical functions, because only few functions are addressed in the phenotype ontologies, nor will this approach provide a high-quality ontology that is suitable for use in applications. Instead, our goal is to extract functions that can be used as the backbone of an ontology of formally defined function categories after a manual review process.

The second major challenge in the extraction of anatomical functions is to provide an analysis and formal representation of the relations between anatomical functions, their bearers and the processes that realize the functions.

### Formal representation of anatomical functions

In our formal analysis, we use the definition of the category *Deafness *in both the Mammalian and Human Phenotype Ontology as an example. The definition in the cross-products of both ontologies is the following statement in the OBO Flatfile Format [20]:

In the OBO Flatfile Format, the definition of an ontological category is started with a [Term] statement, followed by a unique identifier of the category. Everything following an exclamation mark is considered to be a comment.

The GO category GO:0007605 is named "sensory perception of sound" and has a synonym "hearing". The definition of *Deafness *in the two phenotype ontologies we use in our analysis claims that *Deafness *is a process of *HearingP *in which the quality *Absent *inheres. Inherence is a dependence relation between an instance of a quality and the bearer of the quality [21].

There are several problems with the analysis of *Deafness *in the phenotype ontologies. The first problem is that, according to the definition, *Deafness *is a process of *HearingP*. *Deafness *seems to be something different from a process, and certainly different from a *HearingP *process. An absence of hearing means that there is no *HearingP *process what ever properties such a process might have. In particular, *Absent *cannot inhere in a non-existing process and is arguably not a quality at all.

The second problem is that there can be an absence of hearing without there being a case of *Deafness*. In a completely silent environment, both a human or a mouse will experience an absence of *HearingP *even when their *disposition *to hear is present. More precicely, according to the definition of *Deafness*, an absence of sound would also entail *Deafness*.

Therefore, to represent the phenotype *Deafness *formally, we are faced with two challenges: there is an absence of *HearingP *processes and there is also an absence of the *disposition *to hear.

Using our ontological framework for representing malfunctionality, we can represent *Deafness *as the ears' being *malfunctional *with respect to their *HearingF *function. However, a vital point is missing to apply our framework: an ontology of anatomical functions. The absence of such an ontology is one reason for the phenotype ontologies to model abnormal functionings by using processes from the GO.

While the anatomical functions are not yet covered in an ontology, the processes that realize the anatomical functions are present in GO's Biological Process ontology. Therefore, we define a new relation that we call the CC-has-function-realized-by (*hf rb*) relation. This relation is based on the relations CC-has-function and CC-realized-by. The prefix *CC *indicates that the relation takes two ontological categories as arguments. The relations between categories are defined using relations between individuals (*II*-relations), following the pattern of defining CC-relations from the OBO Relationship Ontology [5]. The definition of the relation CC-has-function is given in formula 1, where *E *denotes a category of *Presential*s (in GFO [21]), *Continuant*s (in BFO [22]) or *Endurants *(in DOLCE [23]):(1)

According to this definition, the category *E *has the function (CC-relation) *F *if and only if for every instance *x *of *E *there is an instance *y *of *F *such that *x *has the function (II-relation) *y*.

While the relation CC-has-function follows the standard pattern for defining relations between categories [20], the relation CC-realized-by cannot follow the same pattern. Applying the same pattern would require that, for every function, there is a process that realized the function. Yet, not every function instance is realized, and, according to our considerations about malfunctionality, not every function *can *be realized. Therefore, we have to employ a different definition for the CC-realized-by relation given in formula 2. In the formulation of the definition of the CC-realized-by relation, we assume that functions are not necessarily realized, but when they are realized, then always by processes of a certain kind.

We recognize that this claim is controversial. There may be functions that can be realized by different kinds of processes. However, we assume that it is possible to find a super-category for these kinds of processes that include all and only those process categories that can realize the function. For example, a *TransportF *function will always be realized by *TransportP *processes (yet, arguably, not every *TransportP *process is a realization of a *TransportF *function), and these *TransportP *processes can be of many different kinds, all of which are sub-categories of the *TransportP *process category.(2)

According to this definition, the function category *F *is realized by (CC-relation) the process category *P *if and only if whenever an instance *x *of *F *is realized by some *y*, then *y *is an instance of *P *.

With these definitions of the two relations CC-has-function and CC-realized-by, we can give a definition for the relation CC-has-function-realized-by:(3)

This relation is a connection of the two previously defined relations with an implicit function as argument. The relation CC-has-function-realized-by holds between the category *E *and the category *P *if and only if *E *has the function *F *and *F *is realized by *P *.

The relation CC-has-function-realized-by is a relation between two categories. The relation can be defined in OWL2 as a connection between the two CC-relations by using a property chain:(4)

Such a definition can be used in an OWL ontology in which ontological categories are in the domain of discourse (cf. [21,24,25]), i.e., in which there are OWL classes which have ontological categories as their instances.

A similar connection between the two relations II-has-function and II-realized-by is very different from the relation between the categories: it is a relation between an entity with a function that is in fact (and currently) realized by a process:(5)

### Application to anatomy and phenotype ontologies

We apply the framework for representing functional abnormalities to the automated extraction of anatomical functions from the HPO and MPO. For this purpose, we exploit the naming of the categories in the phenotype ontologies.

We make use of three types of ontologies in our approach:

1. the phenotype ontology that contains abnormal functional phenotypes, either the HPO or the MPO,

2. an anatomy ontology that contains the structures affected by the malfunctionings represented in the phenotype ontology, either the Adult Mouse Anatomy Ontology [5] or the Foundational Model of Anatomy [3], and

3. a process ontology, which contains the processes that realize an anatomical function.

Since functional abnormalities are already classified as subclasses of structural abnormalities in the phenotype ontologies that we consider, we search for a pattern in the phenotype ontologies where

1. a category *C *in the phenotype ontology has a name *name*(*C*); e.g., *Hearing abnormality *(HP:0000364),

2. in *name*(*C*), the name or synonym *name*(*D*) of a GO Biological Process category *D *occurs as a substring and *name*(*D*) is delimited by whitespaces in *name*(*C*); e.g., *Hearing *(GO:0007605),

3. the category *C *is a sub-category of a category *E *with a name *name*(*E*); e.g., *Abnormality of the ears *(HP:0000598),

4. the name *name*(*E*) contains the name or synonym *name*(*F*) of a category *F *from the anatomy ontology and *name*(*F*) is delimited by whitespaces in *name*(*E*); e.g., *Ear *(FMA:52780).

As a consequence, we find abnormalities of GO processes that are classified as sub-categories of abnormalities of anatomical structures in the phenotype ontologies.

To exclude categories that are named after diseases or do not describe abnormalities, we only consider the categories of the phenotype ontologies which contain "abnormal", "impaired", "decreased" or "increased" in their name or synonyms and exclude the rest from our analysis. Furthermore, we excluded the GO categories GO:0032502 (developmental process), GO:0043473 (pigmentation) and GO:0001503 (ossification) from our analysis (see Discussion section).

**Figure 2:**
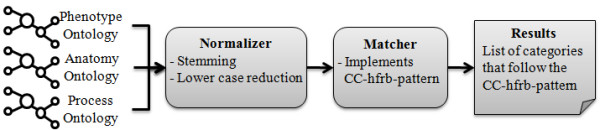
**Processing sequence of the input categories**.

Figure 2 shows an overview of our extraction pipeline. To match the names of the categories, we stemmed all category labels and synonyms in the input ontologies by using the PlingStemmer [26]. The PlingStemmer generates the singular forms of English words. Furthermore, all category labels were reduced to their lower case form before the matching was carried out.

## Results

Using the HPO and the FMA, we could extract 25 structure-process pairs. These pairs and their evaluation are available at our project page. Using the MPO and the MA ontologies, we extracted 331 structure-process pairs. A selection of the pairs which we extracted and which do stand in the **CC-has-function-realized-by **relation is shown in Table 1. In Table 2 we show pairs that do not stand in the **CC-has-function-realized-by **relation. A manual evaluation of our results with respect to the phenotype ontologies showed that we reach a precision of 75% for the HPO and FMA and a precision of 77.27% for the MPO and MA.

**Table 1:** Selection of true positive matches

Structure	Process
cardiovascular system	anatomical structure morphogenesis
uterus	angiogenesis
blood vessel	cell migration
blood	coagulation
female reproductive system	diestrus
reproductive system	fertilization
pancreas	glucagon secretion
mammary gland	lactation

The true positive matches were extracted using the Adult Mouse Anatomy Ontology and the Mammalian Phenotype Ontology.

**Table 2:** Selection of false positive matches extracted from mouse ontologies

Structure	Process
blood	morphogenesis of a branching structure
immune system	t-cell apoptosis
trunk	biological regulation
pancreas	cell differentiation
blood vessel	endothelial cell differentiation

Table 1: The false positive matches were extracted using the Adult Mouse Anatomy Ontology and the Mammalian Phenotype Ontology.

However, these precision values are only valid within the context of the reference ontologies. For example, the **CC-has-function-realized-by **relation holds between *Ear *(FMAID:52780) and *Hearing *(GO:0007605) according to the HPO, although ears only partially contribute to *Hearing*. The quality of the extraction results could be improved by ameliorating the background data upon which the extraction is carried out.

## Discussion

### Functions of parts

Although we successfully applied our proposed ontology pattern to harvest a basic ontology of anatomical functions from the phenotype ontologies by using naming patterns in the ontologies, there are cases in which our pattern yields incorrect results. In particular, the relation between functions and parts of structures remains a topic for further research.

We have argued that an abnormality of a function should be a sub-category of an abnormality of the bearer of the function. However, there may be cases where the bearer of the function is not included in the anatomy ontology or the abnormality of the function bearer is not included in the phenotype ontology. Instead, a structure of which the function bearer is a part or an abnormality of such a structure is included. The functional abnormality pattern is valid if we assume that the abnormality of the part is an abnormality of the whole. This assumption is supported by the phenotype ontologies. Nevertheless, to achieve completeness of both the anatomy ontologies and the phenotype ontologies, and to provide a principled way for building the phenotype ontologies, it is beneficial to include the abnormality of the function bearer whenever an abnormality of a function is included in the phenotype ontologies.

### Text and naming problems

While processing the phenotype ontologies, we discovered several naming problems. First, plural forms are apparently randomly mixed with singular forms of the same term. For example, the label of MP:0003677 is "abnormal ear lobe", while the label of its subcategory MP:0003678 is "absent ear lobes" (plural). The same holds for HP:0000598 (abnormality of the ears) and HP:0000370 (abnormality of the middle ear). We suggest to use the plural form only in the case of explicitly disjunctively defined categories. For example, a category that is defined as the disjunction of the categories "abnormality of the left ear" and "abnormality of the right ear" may be called "abnormality of the ears".

Another difficulty is the mixture of structural and functional abnormalities as category labels. For example, the category HP:0000251 is labeled "abnormality of tear glands OR tear production". This name mixes structural and functional abnormalities: *tear glands *are an anatomical structure, while *tear production *is a process that realizes a function (the function "to produce tears"). To improve the usability and the possibilities for automatic processing of the phenotype ontologies, we suggest a separation of function and structure-based abnormalities. For example, the category HP:0000251 should be split into two distinct categories, one labelled "abnormality of tear glands", the other "abnormality of tear production".

The third issue we found in the phenotype ontologies is the inconsistent use of category labels. The MPO contains the categories "abnormal hearing physiology" (MP:0001963), "hearing disability" and "hearing impairment" (exact synonyms for MP:0001965), "deafness" (MP:0001967) and "impaired hearing" (MP:0006325). The development of a naming convention would not only serve automatic processing of the ontologies, but also help to improve the clarity of the phenotype ontologies.

The current use of polysemous words is one of the main drawback we face when trying to extract functions out of the phenotype ontology. *Ossification*, for example, can be understood both as the process of creating bone tissue and as a property of a bone (the outcome of the process). Thus, while bone ossification relates to the ossification process, skull ossification (HP:0002703) relates to the state of the skull, i.e., the result of the ossification process of the skull. Similarly, *pigmentation *is used widely as a property and not as the process of pigmentation.

Finally, a major problem for the phenotype ontologies is the use of "absent" as a property. In English, "absent" is used as an attributive adjective, and this is one reason why "absent" is present in some ontologies of qualities, in particular the phenotypic quality ontology PATO (PATO:0000462). In most ontologies, such as DOLCE [23], GFO [21] or BFO [22], qualities are dependent on a bearer, an entity *of which *they are a quality. The meaning of "absent", however, entails that there is no such bearer. When "absent" is used in "absent appendix", "absent nipple" or "absent hearing", it does not correspond to an ontological quality [24]. While this fact is increasingly being taken into consideration by the phenotype ontologies in the definition of categories pertaining to the absence of structures, "absent" is still used as a quality in the definition of categories of absent processes (or functions). These categories should be carefully examined and their definition made clear. They can be defined formally by using the functional abnormality pattern [19], which uses a form of the **lacks **relation [27] together with an ontological analysis of functions and dispositions.

The problem of "absent" is not a problem of the phenotype ontologies alone. The PATO ontology also includes "absent" as a quality, and it should be removed from the PATO.

### Ontology problems

Our analysis is hindered by the lack of categories or synonyms for category names in GO's Biological Process Ontology. For example, *tear production*, *cardiac conduction*, *hair pigmentation *or *taste sensation *are not in the GO, yet their existence is indicated by reference to these processes in the phenotype ontologies. An extension of the GO together with a consistent naming of the categories in the phenotype ontologies could improve our analysis and the clarity of the phenotype ontologies.

### The need for an ontology of anatomical functions

One major problem in our analysis is the lack of an anatomical functions ontology. The phenotype ontologies imply that *HearingF *would be a function of the ears, by stating that an abnormality in *HearingF *is a sub-category of *Abnormality of the ears*. However, the ears can be normal and be functioning normally and still there may be an absence of *HearingP*. In particular, *Deafness *may be the result of an abnormality of the ears, or it may be the result of an abnormality in the nervous system. For example, an abnormality in the brain can impair *HearingF *just as well as an abnormality in the ears can. The ears only partially contribute to *HearingP*, and not every abnormality of *HearingF *is an abnormality of the ears. Therefore, the ears and *HearingP *do not stand in the **CC-has-function-realized-by **relation according to our definition: the ears have some function which, if realized, is realized by processes that may be *part *of *HearingP *processes, but are not necessarily *HearingP *processes themselves, nor are they always part of *HearingP *processes. Instead, the function of the ears is realized by processes of the kind *Detection of mechanical stimulus involved in sensory perception of sound *(GO:0050910), which are a **part-of ***Hearing *in the GO.

To prevent this kind of erroneous naming or definition of categories, an explicit relation between anatomical structures and their functions is needed, on which the category definitions in the phenotype ontologies should be based. Such an explicit relation between structures and their functions can be achieved by the introduction of an ontology of anatomical functions and the use of the **CC-has-function **relation, or without the introduction of an ontology of anatomical functions and the use of the **CC-has-function-realized-by **relation.

One advantage of our introduction of the relation **CC-has-function-realized-by **is that a needless duplication of the processes in the GO is avoided. In particular, many functions do not need to be named explicitly because the processes in the GO are defined as processes that realize a given function. A difficulty in hiding the function by using the **CC-has-function-realized-by **relation occurs when one kind of function can be realized by multiple kinds of processes. In this case, a new super-category for all the kinds of processes that may realize the function must be introduced and used as the argument in the **CC-has-function-realized-by **relation. This new category would be defined as the category of all processes that realize a given function - a common form of defining processes.

However, a separate ontology of anatomical functions may provide benefits over indirectly relating structures and processes by using **CC-has-function-realized-by**. With the availability of an ontology of anatomical functions, the inner structure of functions can be represented [14], relations between functions themselves can be established (such as functions that **support **or **prevent **other functions [15]) and properties can be assigned to functions.

### Suggestions for future development of phenotype ontologies

Overall, our analysis of means for extracting and representing functions led to the discovery of several shortcomings of current ontologies. The following list epitomizes these shortcomings and presents suggestions for the future modelling of biomedical ontologies in general and phenotype ontologies in particular.

• Naming conventions: plural and singular form seem to be used inconsistenly in categories labels. For example, MPO contains the categories labeled "abnormal ear lobe" (MP:0003677) and "absent ear lobes" (MP:0003678). We suggest to use the plural form exclusively for naming categories defined disjunctively.

• Mixture of functional and structural abnormalities: several categories are labeled with terms that denote a mix of structural and functional abnormalities. An example for such a mixture is the category label "abnormality of tear glands OR tear production" (HP:0000251). To provide a clear classification founded in ontological principles and to enable automatic processing, we suggest to split such classes into distinct classes: *Abnormality of tear glands *and *Abnormality of tear production*.

• Use of "Absent" as property: previous work has pointed out that *Absent *is not an ontological property (see e.g., [24,27]). We propose the use of a variant of the **lacks **relation instead of *Absent *to improve the clarity of the phenotype ontologies and to enable consistent reasoning on them.

• Missing categories: Some categories implied in the phenotype ontologies are absent in the GO. These categories include *Hair pigmentation *and *Taste sensation*. Adding these categories to the GO would improve the automatic extraction of cross-products to link phenotype ontologies with the GO, and the use of the **CC-has-function-realized-by **relation in the anatomy ontologies provides a method to discover the missing categories in the GO.

• Ambiguous category names: Some terms have been used in literature to denote both processes and states. An example for such a term is "ossification", which can refer to the process of *Ossification *(GO:0001503) or a property of a bone which is the outcome of such a process. To ensure that terms in the phenotype ontology are monosemous, we would suggest the addition of more specific terms to class labels. For example, we would suggest altering the label "abnormal bone ossification" (MP:0008271) into "abnormal bone ossification process" or "abnormal bone ossification state" as required.

• Mapping of parts to functions of whole: Some of the functions of anatomical structures implied by the phenotype ontologies are mappings from a part of a complex anatomical structure to the function realized by the whole of the complex structure. For example, *Hearing abnormality *(HP:0000364) being a subclass of *Abnormality of the ears *(HP:0000598) implies that a function of the ears is realized by *Hearing *processes. Rather, a function of the ears is realized by a part of *Hearing *processes, namely by *Detection of mechanical stimulus involved in sensory perception of sound *(GO:0050910). The use of the **CC-has-function-realized-by **relation in the anatomy ontologies can help to prevent these errors.

## Conclusions

We present an ontology design pattern for the representation of functional abnormalities. The design pattern is applicable to the Human Phenotype Ontology and the Mammalian Phenotype Ontology. We show how to model anatomical functions by using processes from the Gene Ontology that may realize these functions. For this purpose, we introduce a new relation between categories of anatomical structures and process categories. This relation states that an anatomical structure has some function that is realized by a process of a certain kind. Using this relation, functions can be specified without the explicit introduction of an ontology of anatomical functions.

We evaluated our method by exploiting the naming of categories from the phenotype ontologies to extract structure-process pairs that stand in the relation we introduced. We extracted several structure-process pairs from the Mammalian Phenotype Ontology together with the Adult Mouse Anatomy Ontology, and from the Human Phenotype Ontology together with the Foundational Model of Anatomy.

In our analyis, we found several problems with the phenotype ontologies. In particular, we discovered ambiguous namings of the categories and suggest the use of a naming convention for the categories in the phenotype ontologies. Additionally, we found a number of problematic formal definitions of categories in the phenotype ontologies. Most of these are categories of the *malfunctional *type: the loss of the disposition to perform a certain function. The use of our ontological framework would permit an improved ontological representation of functional phenotypes and better capabilities for knowledge extraction from the phenotype ontologies.

## Competing interests

The authors declare that they have no competing interests.

## Authors' contributions

JK, RH and AN conceived of the study, RH and AN formalized, implemented and evaluated the method, JK supervised the project. RH and AN drafted the manuscript. All authors read and approved the final manuscript.
